# Estimating the pre- and post-diagnosis costs of tuberculosis for adults in Pakistan: household economic impact and costs mitigating strategies

**DOI:** 10.1186/s41256-022-00259-x

**Published:** 2022-07-21

**Authors:** Shama Razzaq, Aysha Zahidie, Zafar Fatmi

**Affiliations:** 1 Health Services Academy, Opposite National Institute of Health (NIH), Islamabad, Pakistan; 2 Aga Khan University, Community Health Sciences Stadium Road, PO Box 3500, Karachi, 74800 Pakistan

**Keywords:** Household expenditures, Pre- and post-diagnostic costs, TB care, Adults, Pakistan

## Abstract

**Background:**

Despite free tuberculosis (TB) care in Pakistan, patients still have to bear high costs, which push them into poverty. This study estimated the pre- and post-diagnosis costs households bear for TB care, and investigated coping mechanisms among adults ≥ 18 years in Karachi, Pakistan.

**Methods:**

We conducted a cross-sectional study comprising of 516 TB patients identified with completion of at least one month intensive treatment from four public sector health facilities from two institutes in Karachi, Pakistan. A standardized questionnaire to estimate patient's costs was administered. The study outcomes were direct medical and non-medical costs, and indirect costs. The costs were estimated during pre-diagnostic and post-diagnostic phase which includes diagnostic, treatment, and hospitalization phases. A descriptive analysis including mean and standard deviation (± SD), median and interquartile range (IQR), and frequencies and proportions (%) was employed.

**Results:**

Out of 516 TB patients, 52.1% were female with a mean age of 32.4 (± 13.7) years. The median costs per patient during the pre-diagnostic, diagnostic, treatment and hospitalization periods were estimated at USD63.8/ PKR7,377, USD24/ PKR2,755, USD10.5/ PKR1,217, and USD349.0/ PKR40,300, respectively. The total household median cost was estimated at USD129.2/ PKR14,919 per patient. The median indirect cost was estimated at USD52.0/ PKR5,950 per patient. Of total, 54.1% of patients preferred and consulted private providers in the first place at the onset of symptoms, while, 36% attended public healthcare services, 5% and 4.1% went to dispensary and pharmacy, respectively, as a first point of care.

**Conclusions:**

TB patients bear substantial out-of-pocket costs before they are enrolled in publically funded TB programs. There should be provision of transport and food vouchers, also health insurance for in-patient treatment. This advocates a critical investigation into an existing financial support network for TB patients in Pakistan towards reducing the burden.

## Background

Tuberculosis (TB) leads to poverty and is the second leading cause of mortality worldwide among infectious diseases, resulting in more than one million deaths every year, whereas, most of the TB burden occurs in low and low-middle income countries [1]. The Eastern Mediterranean Region contributed for 8.3% of the most TB cases found in the WHO regions around the globe, whereas, Pakistan ranked 5^th^ among the 30 high TB burden countries and contributed 5.8%, bearing approximately 69% of the TB burden of the Eastern Mediterranean Region (EMR) [2]. The estimated prevalence rate for all types of TB cases was 342 per 100,000 population and the incidence rate was 275 per 100,000 population [3]. The case detection rate for all types of tuberculosis was 64% of estimated new cases i.e. approximately 510,000 new TB cases were reported annually. Extra-pulmonary TB (EPTB) was also increasing and accounted for 15% of the total TB cases [4, 5]. Pakistan’s National TB control program (NTP) adopting directly observed therapy short course strategy (DOTS) aims for adherence to treatment and better treatment outcome to include free first line anti-TB medicines, free or low cost investigations, and no administrative charges [6]. Despite of the free treatment plan, the patients still bear substantial additional costs. These include travel to seek care, loss of earnings due to absence from work, pre-diagnostic investigation costs due to delay or miss-diagnosis, accommodation and food costs for in-patient care, and costs for drug-resistant and extra-pulmonary TB treatment [7].

TB mostly affects the poorest of the poor and substantially contributes to the disease-poverty trap as it has a huge economic impact on households [7]. According to the latest survey of Planning Commission of Pakistan, 24.3% of the population (55 million, approximately) were living below the poverty line during 2015 – 2016 [8]. Out-of-pocket (OOP) healthcare payments were made by 57.6% of households in Pakistan [9, 10]. The majority of people in Pakistan (> 80%) seek health care from the private sector, which puts an extra economic burden on households [6]. Although, a study was conducted recently in Karachi to estimate TB expenditures which has several methodological limitations, thus raising questions about its validity and policy implications of the study findings. Moreover, overall total direct and indirect costs were reported in the study in the form of a mean without standard deviation [11]. Siapka et al. (2020) gathered evidences from low and middle income countries to address the issue of standardized cost of drug susceptible TB care which provided a standardized cost data estimated at country level to prioritize the disease [12]. Lack of standardized methods to calculate the cost and disparities in cost-effectiveness analysis based on different models for TB management strategies resulted in scarcity of accurate TB care cost estimation [13].

Hence, we designed a study to calculate household expenditures including direct and indirect TB care costs for pre- and post- diagnostic (diagnostic, treatment, follow-up and hospitalization phase) periods among adults aged 18 years and older treated in public health facilities in Karachi, Pakistan.

## Methods

### Setting and study design

This cross-sectional study was conducted in four public health facilities from two institutes. One health facility for TB treatment was under Civil Hospital, and rest of the three facilities were working under the Ojha Institute of Chest Diseases under Dow University of Health Sciences which were present at different locations. All four public health facilities were visited by a large majority of TB patients. Most of these patients belonged to low and middle income categories whereas income categories were defined as assets based wealth quintile indicator reported in National demographic health Survey of Pakistan (2017–18) [14].

### Sample size estimation

The sample size in the study was calculated to be 516 participants, based on median costs of USD24.78 i.e. PKR 2,725, and variance of 178.75 [15], and bound by an error rate of 5%, assuming approximately 5% of refusal rate or data incompleteness.

### Questionnaire development

A standardized structured questionnaire titled, “Tool to estimate the patient’s cost”, was developed from the Poverty sub-working group of the Stop TB Partnership [16]. This tool was adapted, modified, translated to the local language, i.e., *Urdu,* and pretested on a small group (n = 25) of participants at the same facilities before distribution and actual data collection. This questionnaire addressed three types of direct and indirect costs: 1. costs for health services during the pre-diagnostic, diagnostic and treatment phases, 2: costs of travel to seek health care and costs for food, and 3. estimated costs in connection with loss of income, travel time, waiting, and seeing health care providers as well as loss of productivity due to absence from work [17].

### Cost estimation

Costs were estimated for each phase separately, i.e. the pre-diagnostic phase when the patient was symptomatic but not yet diagnosed and the post-diagnostic phase when the patient was diagnosed, had treatment and follow-up. Cost details are provided in Table 1. Direct medical and non-medical costs per patient were calculated by multiplying the costs (e.g. administrative, investigation or medication costs) with the frequency of patient’s visit to the health facility. Indirect costs were calculated by tabulating the total number of hours commuting to and spent in a healthcare setting. Taking into account an average working day of 8 h, the monthly household income per patient reported by the patient was used to derive an hourly wage rate which ranged from USD0.5 to USD2 to determine the indirect costs [18]. All cost data were collected in PKR and reported in USD and PKR both (Mid Exchange Rate as of 2017: USD 1 = 115.5 PKR).

**Table 1:** Operational definitions for cost categories:

Variables	Definitions
Direct medical and non-medical cost	Out-of-pocket expenditure for TB treatment such as consultation, investigations, travel and food costs
Indirect cost	Income loss of patient because of absence from work due to TB and/or time to seek care (includes time for travel, waiting and consultation and/or during hospitalization)
Pre-diagnostic costs	Costs incurred during the phase from onset of symptoms till the diagnosis of TB
Post-diagnostic costs:	It includes diagnostic, treatment, follow up and hospitalization costs
Diagnostic costs	Costs incurred by patient and household for diagnosis
Treatment costs	Costs related to DOTs, fetching medicines from health facility and costs related to any other medicines such as multivitamins
Follow-up costs	Costs associated with investigation, any other medication, and travel and food costs during follow up visits
Hospitalization costs	Costs for hospitalized patients including treatment, administrative (bed), laboratory investigation and medicine, food and travel costs and loss of income due to absence from work

### Data collection and processing

We trained a field team for the data collection who invited the participants, obtained an informed consent and administered the questionnaire and interviewed them which took around 25 to 30 min per participants. Both male and female patients ≥ 18 years and diagnosed with TB (pulmonary or extra-pulmonary) who had experienced at least 1 month of treatment in the intensive phase were recruited by the team from August 2017 till September 2018 by using a non-probability consecutive sampling method [19]. After completing the data collection, data was entered twice into EpiData v3.1 for double data check and then exported to SPSS version 21 for cleaning and analysis. The data were explained by using proportions for categorical variables, mean (standard deviation, SD) for continuous variables, and median (interquartile range, IQR) was calculated for costs related data for all phases of TB management.

### Ethical approval

The Ethics Review Committee of Aga Khan University provided the approval for study (ERC# 4026-CHS-ERC-16). The Institutional Review Board (IRB) of Dow University of Health Sciences also approved the study (IRB-679/DUHS/Approval/2016/152). Prior to the interview, the purpose and nature of the study was explained and written informed consent was obtained from each study participant.

## Results

### Socio-demographic characteristics

Among TB patients aged ≥ 18 years, 269 (52.1%) were females; 382 (74.0%) were or had been married, and 32.1% belonged to most common ethnicity living in the city, i.e., Urdu. The mean age of the participants was 32.3 (± 13.7) years. 215 (41.7%) never attended school and 221 (42.8%) were employed. The mean household income was 17,453 PKR (± 15,192PKR). One hundred thirteen (21.9%) were living in a highly crowded household, with more than 5 members per room. The characteristics are summarized in Table 2.

**Table 2:** Socio-demographic characteristics of adult (≥ 18 years) patients at public healthcare facilities with TB care in Karachi, Pakistan (n = 516)

Characteristics	n = 516 (%)
*Age in years* [(Mean (SD)]	32.35 (13.7)
*Gender*	
Male	247 (47.9)
Female	269 (52.1)
*Marital status*	
Never Married	134 (26.0)
Ever Married	382 (74.0)
*Ethnicity*	
Urdu	165 (32.1)
Punjabi	91 (17.6)
Sindhi	103 (19.9)
Pushto	83 (16.1)
Baluchi	74 (14.3)
*Religion*	
Muslims	492 (95.3)
Non-Muslims	24 (4.7)
*Education*	
Didn’t attend school	215 (41.7)
Madrassa only	18 (3.5)
Primary class	73 (14.1)
Secondary class	154 (29.8)
Intermediate	41 (7.9)
Graduation	15 (2.9)
*Employment status*	
Employed	221 (42.8)
House lady	202 (39.1)
Unemployed	30 (5.8)
Student	30 (5.8)
On sick leave	27 (5.2)
Retired	6 (1.2)
*Household income* in PKR (mean[SD])	17,453 (15,192)
*House ownership*	
Own	275 (53.3)
Rented	241 (46.7)
*Crowding index*	
≥ 5 Members per room	113 (21.9)
< 5 Members per room	403 (78.1)

### Health-seeking behavior and TB profile

279 (54.1%) patients first visited a private health facility as a first point of contact after the onset of illness. 256 (49.6%) were pulmonary smear + ve; 112 (21.7%) were smear –ve; and 148 (28.7%) were extra-pulmonary TB cases. Patients under treatment during the continuous phase were 375 (72.7%). Contact screening of study participants’ family members was not done of nearly all 472 (91.5%) participants. A summary of the TB profiles is presented in Table 3.

**Table 3:** Description of type of TB and its treatment, health seeking among adult (≥ 18 years) patients at public healthcare facilities with TB care in Karachi, Pakistan (n = 516)

Characteristics	n = 516(%)
*Type of TB*	n (%)
Pulmonary, smear + ve	256 (49.6)
Pulmonary, smear -ve	112 (21.7)
Extra pulmonary	148 (28.7)
*Treatment regimen*	
Cat I	400 (77.5)
Cat II	116 (22.5)
*Type of patient*	
New	400 (77.5)
Relapse	70 (13.6)
Treatment after default	19 (3.7)
Treatment after failure	19 (3.7)
Transfer-In	03 (0.6)
Treatment after loss to follow up	05 (1.0)
*Treatment phase*	
Intensive phase	141 (27.3)
Continuous phase	375 (72.7)
*Total duration of planned treatment*	
6 months	307 (59.5)
8 months	118(22.7)
> 8 months	91(17.8)
*No of contacts screened*	
None screened	472 (91.5)
≥ 1 member screened	44 (08.5)
*First contact for seeking TB care*	
Private hospital/clinic	279 (54.1)
Government hospital	186 (36.0)
Dispensary	30 (5.8)
Pharmacy/drug store	21 (4.1)

### Pre-diagnostic costs and Post-diagnostic costs

Before diagnosis, TB patents incurred a median (IQR) cost of USD63.8 (44.7–90.5). Direct medical and non- medical costs amounted to median (IQR) USD25 (12.1–35) and USD5.2 (4.3–6.5). Indirect cost amounted to median (IQR) USD 29.2 (19.5–47) (Table 4).

**Table 4:** Summary of direct and indirect median costs [IQR] associated with TB care among adult (≥ 18 years) patients at public healthcare facilities with TB care in Karachi, Pakistan (n = 516)

Total costs		Direct medical costs		Direct non-medical costs		Indirect costs	
USD	PKR	USD	PKR	USD	PKR	USD	PKR
Costs summary for outpatient care (n = 516)							
*Pre-diagnostic costs (median costs [IQR])*							
63.8 [44.7–90.5]	7377 [5168–10450]	25 [12.1–35]	2875 [1400–4000]	5.2 [4.3–6.5]	600 [500–750]	29.2 [19.5–47]	3375 [2250- 5400]
*Diagnostic costs (median costs [IQR])*							
24 [15–35.5]	2755 [1686–4095]	8.0 [0.3–18.2]	900 [40–2100]	4.3 [3.0–12.1]	500 [350–1400]	6.5 [4.0–9.5]	750 [450–1100]
*Treatment costs (median costs [IQR])*							
10.5 [6.5–15.6]	1217 [750–1800]	0.00 [0 – 0]	0.00 [0–0]	3.1 [1.7–5.5]	355 [200–637]	6.0 [3.5–10.1]	698 [400–1170]
*Follow up costs (median costs [IQR])*							
15.7 [8.8–32.4]	1816 [1020–3747]	2.7 [1.3–9.0]	315 [150–1035]	3.5 [1.3–7.8]	400 [150–900]	4.9 [2.6–8.7]	561 [300–1000]
*Total household costs (median costs [IQR])*							
129.2 [99.0–172.2]	14,919 [11415–19892]	42.0 [26.4–68.4]	4835 [3050–7895]	22.8 [15.6–3.63]	2635 [1800–4187]	52.0 [36.1–72.0]	5950 [4172–8304]
*Costs summary for Inpatient care (n* = *91)*							
349.0 [147.2–463.2]	40,300 [17000–53500]	284.0 [121.2–450.2]	32,800 [14000–52000]	56.3 [23.4–130.0]	6500 [2700–15000]	8.7 [4.7–18.2]	1000 [540–2100]

After diagnosis, TB patients incurred a median (IQR) cost for treatment and follow-up of USD 10.5 (6.5–15.6) and USD 15.7 (8.8–32.4), respectively (Table 4).

The total household median (IQR) cost was estimated to be USD 129.2 (99.0–172.2). Whereas, in-patient costs amounted to median (IQR) 349.0 (147.2–463.2). Out of total, 17.6% of the patients were hospitalized during treatment with mean (± SD) length of stay during hospitalization was 6.5 days (± 3.2 days) (Table 4).

Additionally, the laboratory investigations (Xpert, liver function tests (LFTs), complete blood count (CBC)) costs amounted to median (IQR) USD 11.6 (7.8–35.4 and non-TB drugs costs amounted to median (IQR) 9.5 (5.5–14.7).

### Coping mechanism

**Fig. 1:**
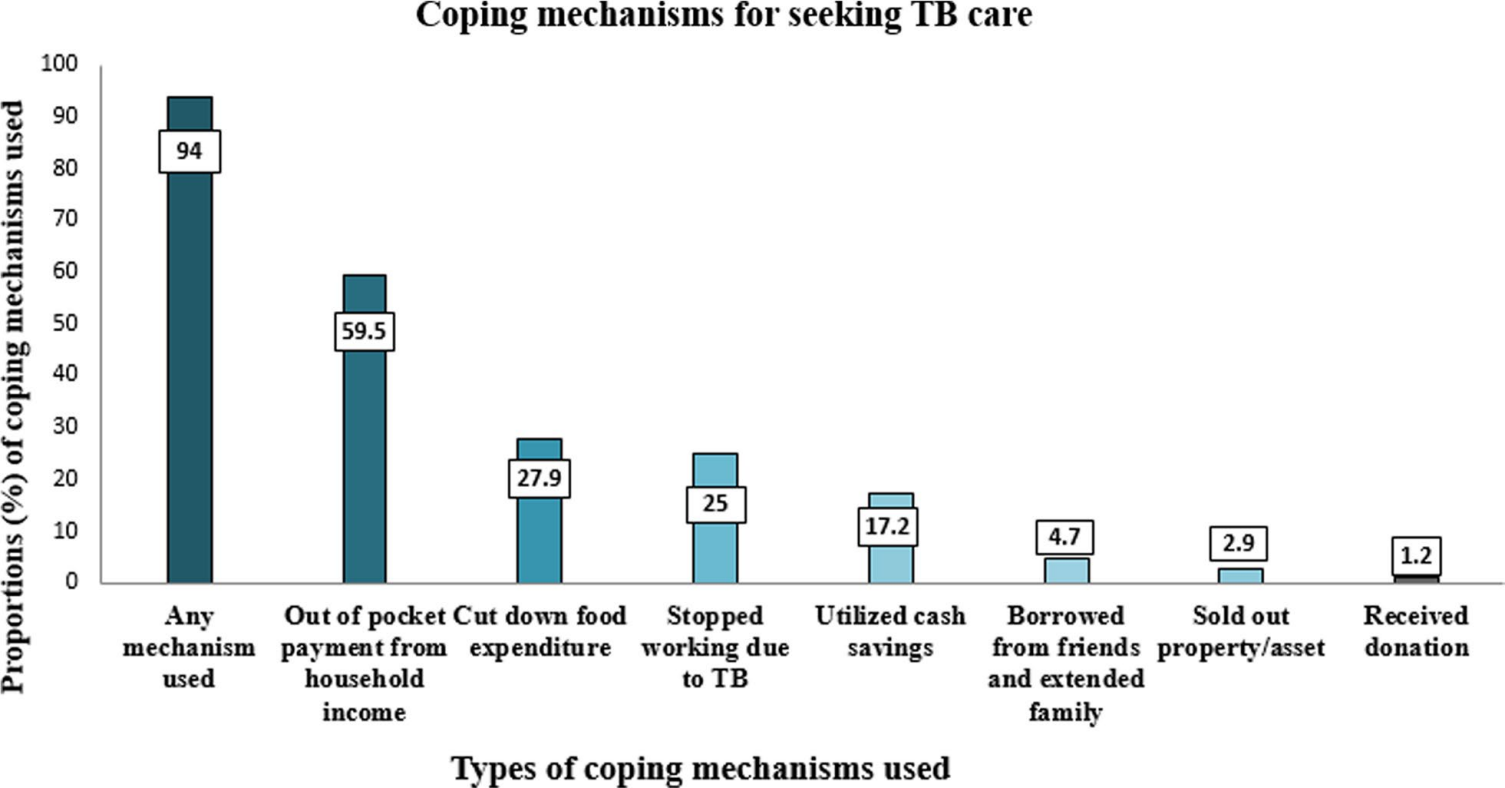
Summary of coping mechanisms among study participants (≥ 18 years) attending public healthcare facilities with TB care in Karachi, Pakistan (n = 516)

For the purpose of seeking TB care, 59.9% of the patients spent out of pocket from household income followed by 27.9% who cut down food expenditures, 25% who stopped working due to TB, and 17.2% who utilized cash savings. (Fig. 1).

### Contribution of each costs category during different phases of TB Care

**Fig. 2:**
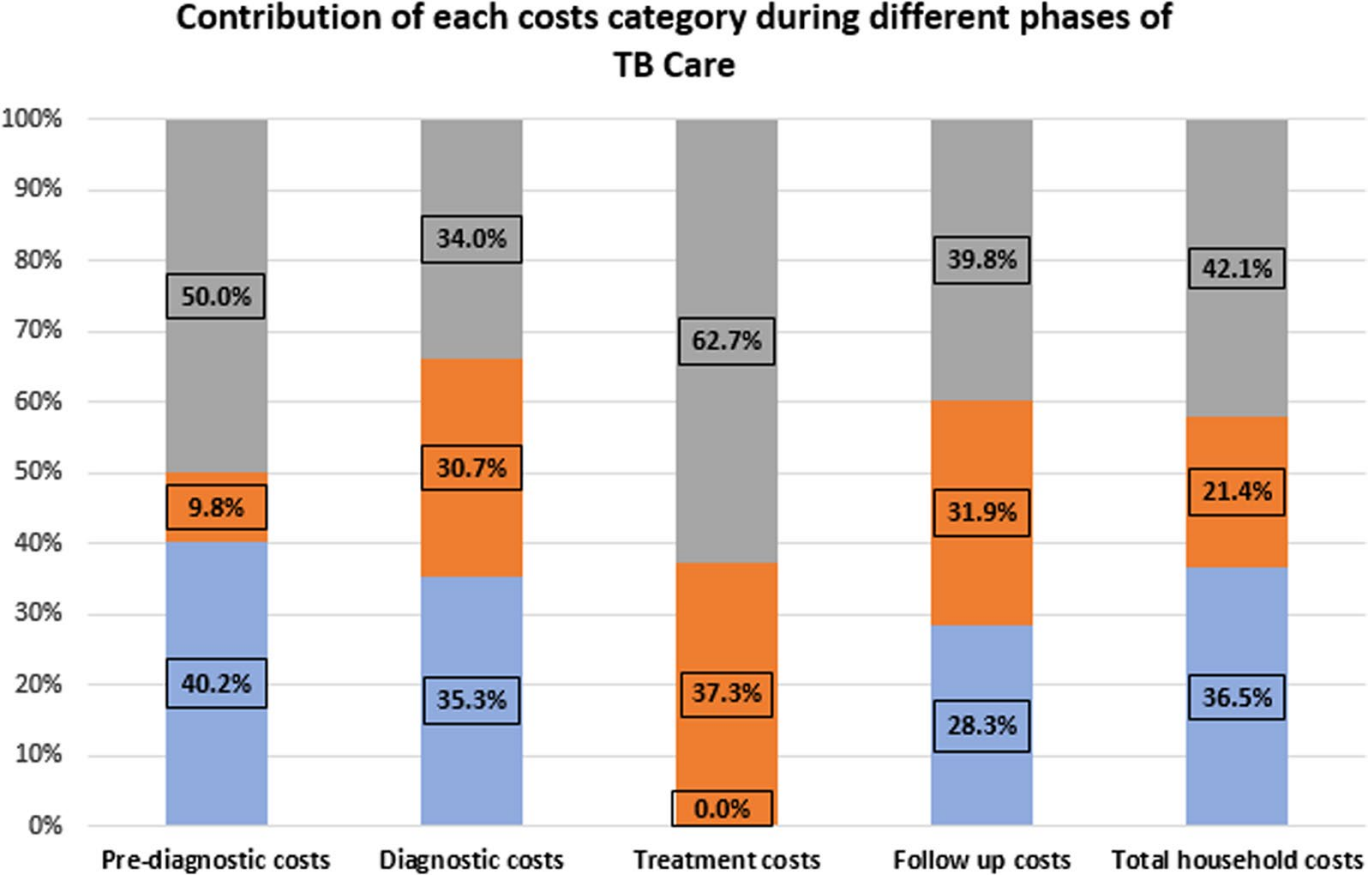
Contribution of each costs category during different phases of TB care among study participants (≥ 18 years) attending public healthcare facilities in Karachi, Pakistan (n = 516)

Out of total household costs, indirect costs shared the most i.e. 42.1%, followed by direct medical costs i.e. 36.5% and direct non-medical costs i.e. 21.4%. During the pre-diagnostic phase, indirect costs shared 50% and direct medical costs shared 40.2%. During the diagnostic, treatment and follow-up phases, indirect costs was 34%, 62.7%, and 39.8%, respectively, while the direct non-medical costs was 30.7%, 37.3% and 31.9%, respectively (Fig. 2).

## Discussion

This is one of the few studies reporting the cost effect of tuberculosis during different phases of TB management and calculated the economic burden associated with TB in Pakistan. This study revealed that substantial financial costs incurred across pathways to TB care by patients and their families with a significant burden of TB and poverty and where basic TB services are free of charge such as consultation fee, AFB sputum smear, chest X-ray in Pakistan. The total median (IQR) household cost for TB care per patient, including both direct and indirect costs, amounted to USD129.2 (99.0–172.2)/ PKR14,919. Overall, our study estimate is relatively comparable to a total median cost amounting to USD171 (75.6—387) ascertained in a population in China [20]. But our cost estimates were found to be comparatively lower total median costs than those reported in Ghana, Viet Nam and the Dominican Republic i.e. USD202, USD758 and USD742, respectively [21]. This could be possibly due to a heavy reliance on out of pocket expenses and inequality in healthcare distribution in low and middle income countries [12].

This study estimated indirect median costs amounting to USD52.0 (36.1–72.0)/ PKR5950 which is a significant contributor (42.10%) of the total household costs for TB care. This finding is comparable to a study conducted in a neighborhood which found estimated median indirect costs at USD78 [20]. A study conducted in Nigeria reported that indirect costs per patient was USD79.13, which is relatively comparable to our study estimates [22]. Furthermore, looking at the pattern of costs contribution during different phases of TB care (Fig. 2), it is evident that indirect costs are relatively higher than other cost categories during different phases and it is the highest contributor during treatment phase i.e. 62.7%. Our finding for higher indirect costs throughout is relatively comparable to the finding in India which estimated that the contribution of indirect costs (loss of income) incurred by patients for TB management was 41% out of total costs [23]. The indirect costs could be due to absenteeism from work and loss of productivity and income while suffering because of long course of disease and recovery pathway. Among Thai adults, a study reported that patients experienced highest indirect costs out of total expenditure due to the patients’ reduced ability to work and loss of income [24]. The policy implication to curb the higher indirect costs and loss of income might be an assistance of TB patients with leave compensation with introducing a paid sick leave package along with the flexibility in working hours. Additionally, the social mobilizers or health workers can dispense medications among TB patients at home, ultimately reducing indirect costs incurred while travelling and waiting.

The substantially higher median (IQR) cost during the pre-diagnostic phase reported identified in the study amounted to USD63.8 (44.7–90.5)/ PKR7,377, contributing 49% of the total household out of pocket payment for TB care. This could be due to informal care such as self-treatment and the practice of seeking health care from private facilities, which demand sizeable amounts of direct medical costs compared to public facilities where medical costs are minimum and more tolerable. In our study, patients sought care from private hospitals (54.1%), dispensaries (5.8%), and drug stores (4.1%) when first contracting TB. The higher cost during pre-diagnostic phase was also reported in Ghana, Viet Nam, Dominican Republic and Ethiopia [21, 25]. Indirect median (IQR) cost in pre-diagnostic phase was USD29.2 (19.5–47)/ PKR3,375 which has a significant contribution i.e. 50% out of total pre-diagnostic cost. Indirect costs have also been higher and contributed significantly as shown in earlier studies [21]. Since, the quality care in affordable price rates has remained worst at public health care facilities in Pakistan. Therefore, the policy implications for increasing utilization of public health care services are to introducing and regularizing quality facilities so that people attend them as a first point of contact with confidence and advocate for those affordable quality facilities. Hence, it will ultimately reduce the higher pre-diagnostic costs reported in our study due to utilization of private services at first place by majority of patients.

Diagnostic Median (IQR) costs were reported in this study at USD24 (15–35.5)/ PKR 2,755 where direct medical costs shared 35.3% of it, which is significant. A longitudinal study conducted in Ethiopia assessed the pre and post-diagnostic cost of TB reported median costs for diagnostic phase amounting to USD35.02 [25]. Despite free AFB sputum smear and Chest X-ray, our study reported diagnostic cost which might be due to the laboratory investigations from private laboratories since people tended to have low confidence on the quality of services provided by public providers as training provided for TB case management for the public providers and private providers is different.

Besides the direct medical costs, indirect median (IQR) costs during the diagnosis phase reported in this study shared higher contribution USD 6.5 (4.0–9.5)/ PKR750. A comparable estimate of indirect median costs amounting to USD9.2 was reported in Ethiopia [26]. This might be because of time taken off from work for diagnostic tests and visits to receive the final diagnosis and for the prescription of treatment accordingly. During this phase, the patient either takes sick leave or resigns depending on the severity of illness, either way facing loss of income.

During the treatment and follow-up phases, the median (IQR) costs reported were USD10.5 (6.5–15.6)/ PKR1,217 and USD15.7 (8.8–32.4)/ PKR1,816, respectively. The cost, in contrast, is very much lower to the estimated cost reported in Ghana was USD429.6 [27]. Despite free TB medications and consultation, our study estimated a treatment and follow up cost which might be due to the contribution of an indirect costs i.e. 62.70% and 39.8% during treatment and follow-up duration, respectively, as patients or the guardians had to travel to the facility to pick up the drug. Additionally, the follow-up cost in this study is slightly higher than the treatment costs since follow up visits require laboratory investigations to determine progress towards recovery which are not covered under “Free TB care”.

Patients hospitalized once ever during the course of treatment were 17.6% (n = 91). This is less than the 33% and 23% reported in Ghana and Viet Nam [21]**.** Estimated hospitalization median (IQR) costs in our study amount USD 349.0 (147.2- 463.2)/ PKR40300. This estimate is considerably higher compared to findings reported in Ghana and Viet Nam which amounted to USD42 and USD118, respectively [21]. The possible reason for the higher costs reported among hospitalized patients in our study might be the severity of symptoms which could be associated with a relative delay in seeking care, i.e. after five to six weeks, hence, a late diagnosis could lead to an advanced stage of the disease and turn into complications [28–30]. Another reason could be the difference in costs of providing care from the provider’s perspective which might vary from one country to the other. The policy implications to reduce in-patient costs would be at programmatic level with the identification of gaps in the current national TB control program for addressing costs of TB illness and addressing them timely. In our study, a major burden is the cost of hospitalization, food and transport and loss of income. TB care support by the program may consider insurance coverage policy for in-patients and food and transport vouchers to reduce direct non-medical costs. Out-of-pocket payments for tuberculosis (TB) care among adults in Pakistan cause substantial financial burden for households despite of “Free TB care” policy. Overall, the global evidences also found that the financial burden has been pushing families into a catastrophic condition which suggested that the policy implications at a global level should be based on socioeconomic support through cost cutting initiatives, having a national data base for determining household poverty in order to enroll them into a social health security schemes [31–33]. Therefore, policy makers are required to imply an inclusion of social support for TB care in currently functioning social health protection “Sehat Sahulat Program”, a safety net program for poor in Pakistan. The policy makers in coordination with National TB control program should encourage the basic assessment of socio-economic status of TB patients for including them in the safety net program. The example has been emerging such as expansion of the Ghana’s National Health Insurance scheme for TB patients to provide protection financially through social protection schemes [34]. Additionally, non-governmental organization (NGOs) and philanthropists can support national program linking household poverty within community which would help in restructuring program in relation to the TB care expenditure. They can also assist in increasing awareness through mass media campaigns helping in reducing taboos, assuring timely diagnosis, providing effective treatments and reducing bacterial transmission through unidentified and untreated cases.

This study also reported common coping modalities which patients and their families used to afford expenditure for TB care. Patients and their families have to arrange money to bearing high costs during pre-diagnostic phase while availing private services, high direct non-medical costs and indirect costs during post-diagnostic phase. Therefore, a substantial proportion of participants i.e. 94% used any mechanism to finance the TB care thus further pushing their family and households into more poverty. Further, this study reported the distribution of different mechanisms to bearing the cost of TB care; 59.5% spent out of pocket from their income, 27.9% cut down food expenditure, 25% stopped working due to TB, 17.2% utilized cash savings, 4.7% borrowed money from friends and extended family, 2.9% sold out their assets, and 1.2% received donations. In previous study conducted in Ethiopia reported that working hours by TB patients were reduced by 78% due to TB and the illness forced them to stop working [35]. Evidence from Bangladesh and China also suggested that borrowing and selling are used as coping strategies to meet the health care related expenditure [36, 37].

This study has some strengths. The study is unique in Pakistan in reporting TB expenditure estimates in detail, comprising of costs incurred in different phases such as pre-diagnostic, diagnostic and treatment along with estimating indirect costs (loss of productivity). In addition, this study used a standardized questionnaire specifically developed for estimating patients’ costs for tuberculosis care comprehensively covering all aspects and phases for expenditure. This is among the few studies conducted on cost estimation for treatment of TB patient. The study sample was statistically calculated to determine the median costs from a regional study.

Some limitations of the study need to be kept in mind while interpreting the costs. First, this facility-based study which was conducted in four public sectors where the majority of participants come from low and middle socio-economic groups. However, some patients who utilized the private sector and those unable to visit even the public sector hospital due to poverty are not included in this study. Second, this study was carried out in an urban setting where the socio-economic and socio-demographic status is different (may be higher) than in rural areas. Third, questions about costs and income are subject to recall bias and seasonal fluctuation, where validation of costs is difficult, particularly for those who had a number of visits to hospitals during pre-diagnosis and for those who required retreatment. Fourth, although patient cost surveys in different parts of the world somehow showed similar estimates, costs cannot be compared directly due to different methodologies employed in other studies and costs ascertained at different time periods. Fifth, the cost has been calculated based on a prevalent approach hence, lifetime cost of TB could not be determined.

## Conclusions

TB patients bear substantial out-of-pocket pre-diagnostic and diagnostic costs even before they are enrolled in TB programs. After enrollment in public sector TB programs they still bear a substantial burden of direct non-medical costs. Furthermore, for those TB patients who require admission, the in-patient cost can be overwhelming. TB patients from low and middle income countries require adequate support for diagnosis, and introducing risk protection mechanism via safety net insurance initiatives or cash transfers for direct non-medical and in-patient costs coverage. Additionally, low and middle income countries should have a national data base for determining household poverty in order to enroll them into a social health security schemes. This work should serve as a strong encouragement to revisit the financial support network provided for TB patients and understand the need of introducing social health protection mechanisms in Pakistan for poor.

## Data Availability

The data analyzed during the current study will be available from the corresponding author upon reasonable request. All data generated or analyzed in this study are contained in this published article.
